# Nursing students’ perspectives on learning electronic health record documentation in an academic setting and during clinical placement: a qualitative study

**DOI:** 10.1186/s12912-025-03320-5

**Published:** 2025-07-01

**Authors:** Inger Åse Reierson, Randi Garang Mofossbakke, Hilde Solli

**Affiliations:** 1 Department of Nursing and Health Sciences, Faculty of Health and Social Sciences, University of South-Eastern Norway, Post Box 4, Borre, 3199 Norway; 2 Research Group Clinical Competence in Nursing Education, University of South-Eastern Norway, Porsgrunn, Norway

**Keywords:** Academic electronic health records, Clinical placement, Documentation, Electronic health records, Nursing education, Nursing students, Simulation

## Abstract

**Background:**

Nursing documentation is pivotal for ensuring high-quality nursing care and patient safety. Studies have demonstrated the importance of learning electronic health record documentation in academic settings to prepare nursing students for practice and further training during clinical placement. Training using simulated electronic health records is therefore encouraged. This study aimed to explore bachelor nursing students’ perspectives on learning electronic health record documentation in an academic setting and during their first clinical placement. The study was conducted at a Norwegian university.

**Methods:**

A qualitative approach with an explorative and descriptive design was chosen. Twenty-one bachelor nursing students participated. All had completed a compulsory academic electronic health record documentation course and their first clinical placement, in a municipality nursing home using the same electronic health record system as in the academic setting. Data were collected via focus groups. A qualitative and reflexive thematic analysis was applied.

**Results:**

Two main themes were revealed: (1) Basic learning of electronic health record documentation, with the following subthemes: need for sufficient teacher guidance in the academic setting and appreciation of peer learning during group assignment. (2) Experiences and challenges during clinical placement, with the subthemes: appreciation of mentoring whilst in clinical placement, feeling of being ‘on your own’, worrying about patient safety and reflection on the two-sided learning process.

**Conclusion:**

This study revealed gaps and inaccuracies in the learning of electronic health record documentation during clinical placements. Nursing students emphasized the importance of learning electronic health record documentation in an academic setting as well as having feedback in the clinical learning process. The study suggests improvement in nursing students’ education in electronic health record documentation, especially in clinical settings.

**Supplementary Information:**

The online version contains supplementary material available at 10.1186/s12912-025-03320-5.

## Background

Electronic nursing documentation has been found to improve patient safety and nursing care [1] and is an integral part of the electronic health record (EHR). The EHR refers to an electronic documentation system that provides a thorough and unified presentation of the patient’s health status according to law regulation [2, 3]. In Norway, the documentation of nursing care has been mandatory since 1999 [4] and, since 2019, it must be recorded electronically [2]. According to Norwegian law, nursing students are defined as healthcare workers and are thus obligated to document nursing care performed during their practice periods [4, 5]. Providing learning opportunities for nursing students’ to develop competence in nursing documentation is therefore fundamental for providing high-quality nursing care and securing patient safety [6].

Previous research found that newly-graduated nurses lacked competence in nursing informatics, including EHR documentation [7]. This is supported by recent research, which further points out that a certain level of EHR competency is necessary to provide safe and patient-centred care [8, 9]. Insufficient proficiency in EHR among newly graduated nurses adds to their workload, contributing to increased stress, cognitive overload, and a heightened risk of burnout [8]. Significant gaps and limitations are emphasized in the education in EHR documentation at the undergraduate and graduate levels, posing challenges for nursing students to develop required documentation competencies in practical placements [9]. The clinical placements of nursing students aim to provide experience in executing EHR documentation, enabling them to acquire adequate documentation competence. However, training opportunities for EHR documentation during clinical placement may be limited, especially due to a lack of access to these systems [10]. Several studies have therefore recommended integrating simulated EHR documentation into undergraduate nursing education [6, 9, 11–16]. However, a recent review on nursing students digital health education and training, found that only two of the 36 included studies, reported from EHR learning, both focusing on simulated EHR learning [17].

Training in an academic setting encompasses a simulation-based learning (SBL) approach, defining simulation as ‘a technique that creates a situation or environment to allow persons to experience a representation of a real event for practice, learning, evaluation, testing, or to gain an understanding of systems or human actions’ [18]. Simulated EHRs in an academic setting prior to clinical placement aim to enhance the preparedness of nursing students to apply EHR documentation during internship [19]. It is also associated with bridging the theory– practice gap [20, 21]. Aligning theory and practice are found to improve the students to transfer knowledge to clinical practice [20]. The integration of an EHR system into the nursing curricula that students will meet in the local health setting is recommended [9]. Even though the nursing education takes place in both an academic setting and during practical placements, research on EHR documentation was mostly reported from stand-alone courses either integrated into clinical courses, fundamental courses or as a clinical course [17]. However, recent research found that few educational programs included simulated EHRs in their curricula [14]. Support to nursing students and faculty members in effectively implementing this modality in nursing education is important [22].

To the best of our knowledge, from the last fifteen years no studies have explored the nursing students’ perspectives on learning EHR in the academic setting and consecutively in their subsequent internship. To incorporate such viewpoints, a university in Norway decided to investigate the perspectives of nursing students on learning EHR documentation in the academic setting and subsequent clinical placements during the first year of their bachelor nursing degree programs.

Sociocultural learning theory defines the Zone of Proximal Development (ZPD), as the space where a more skilled person can enable an unskilled person to stretch towards their learning limits [23]. Examples of such skilled people can be teachers, contact nurses in clinical placements or fellow students. Feedback is important for learning [24, 25], and receiving such input from a more experienced person can enhance student learning. The World Health Organization (WHO) [25] highlights that one core competency for nursing educators is to provide timely, constructive, and considerate feedback, aligning with the student-centred pedagogy promoted in higher education by the Bologna process [26].

The Bologna Process also advocates for student involvement in course design, curriculum shaping, evaluation [26], linking such feedback to student-centred learning and curriculum improvement. In simulation research, providing student perspectives has been emphasized to enhance SBL curricula [27, 28]. This is in line with broader trends in higher education that highlight the value of student perspectives in enhancing program quality [29, 30].

## Methods

### Aim

The aim of the current study was to explore bachelor nursing students’ perspectives on learning EHR documentation in an academic setting and during the first clinical placement of the bachelor nursing degree.

### Design

The study used an explorative and descriptive qualitative research design [31]. This design is appropriate when a phenomenon is not fully understood and needs further clarification [31] and aims to produce descriptions close to the data presented by participants [32, 33]. The design was suitable for this study due to the scarcity of in-depth knowledge on nursing student perspectives on learning EHR documentation in academic and during clinical settings.

### Setting

The nursing students attended a compulsory EHR academic course explicitly incorporated into the curricula of a five European Credit Transfer and Accumulation System (ECTS) simulation course focusing on basic nursing care. Before the EHR course, students had completed theoretical lessons on the nursing process, basic nursing care, anatomy, physiology, microbiology and hygiene. Information on the EHR course outline was made available on the university’s digital learning platform. A dummy version of the EHR software used in the students’ impending municipal clinical practice was available via the university’s data platform. Nursing documentation in Norway can either be standardised or composed in free text [3, 34–36]. The EHR system reported in this study is one of the primary systems utilised by Norwegian municipalities [35] and organises free-text nursing documentation into systematized categories [34].

The compulsory academic EHR course started with a three-hour, teacher-led introduction to the simulated EHR software at a data lab, during which the students documented a nursing care plan for a patient from a common case. After the introduction in the data lab, students worked in assigned learning groups on a new case study to develop an EHR nursing care plan. The nursing care plans included data collection - organised into four categories based on human needs: physical-, mental-, social- needs, and also those related to identity and values - and formulation of nursing diagnoses, goals and interventions. Each learning group could utilise a one-hour teacher guidance session while working on the assignment. The nursing care plan was submitted as a group assignment. The course included a class presentation detailing theoretical rationales for the identified and formulated nursing diagnoses, goals, and interventions. The students were also instructed to reflect on their challenges using the EHR system.

The clinical placements were performed after the EHR course. Each student was assigned an individual contact, a registered nurse, as a mentor during clinical placement. This nursing home practice focused on basic nursing care and included EHR documentation.

### Recruitment and participants

A purposive sample was chosen [37]. This sampling approach is often chosen in qualitative descriptive research to include participants who can provide rich data of the phenomenon of investigation [32]. The inclusion criterium in our study was therefore that the participants had attended the EHR course in an academic setting and completed their first clinical placement, in a municipality nursing home using the same EHR system as in the academic setting. Both full-time first-year and part-time second-year nursing students were recruited. Both groups had undergone the same academic courses ahead of the academic EHR course. A total of 59 students were asked to participate, of which 21 accepted, 14 of whom were full-time students, while 7 were part-time students.

### Ethical considerations

The Norwegian Centre for Research Data (NSD) website in February 2018, stated that the study would not involve personally identifiable or any other sensitive data, and that the focus groups would be audio-recorded. This was prior to the General Data Protection Regulation (GDPR) [38] being implemented in Norway, July 2018. Before this, data privacy in Norway was regulated by the Personal Data Act of 2000 [39], which nationally implemented EU Directive 95/46/EC [40]. At this time there was no consensus as to whether audio recordings were considered a form of personal data. As such the response to the preliminary form on the NSD website was that the project did not need to be registered within NSD. However, all the data in the project was stored according to university guidelines and legal rules for anonymity. The study project was approved in writing by the departmental dean. Information about the study was communicated to the students in class and in writing via the university’s digital learning platform to secure informed consent and that the students could withdraw from the study at any time without consequences for their student situation. The students who chose to participate provided written consent prior to enrolment. The study was conducted in accordance with the Declaration of Helsinki and ethical guidelines for research [41].

### Data collection

Focus group interviews were chosen to collect data as these interviews provide the opportunity of participants to share, discuss and reflect upon both their own and other focus group participants’ views on a specific topic, which again can lead to new perspectives [42, 43]. This synergy can produce rich data on the phenomenon under investigation [32]. A semi-structured interview guide was developed. The questions focused on the students’ learning experiences in both an academic setting and also during clinical placement (Supplementary File 1).

The 21 participants had performed their clinical placements in six different nursing homes, each of which used the same EHR system as in the academic setting. They were divided into three focus groups (FGs). Each FG consisted of between six and eight participants. The FG interviews occurred 1 week after the clinical placement was completed, in May 2018, for full-time students (14 students), and in December 2018, for part-time students (7 students) and took place at the university. The FG interviews were conducted by experienced nursing educators familiar with being moderators. None of these educators had been involved in the compulsory EHR documentation course or as supervisors during clinical placements. During the FG interviews, only two moderators and the students were present. The FG interviews were audio-recorded and lasted for between 53 and 60 min. Interviews were transcribed verbatim using an external transcriber.

### Data analysis

A qualitative, reflexive thematic analysis was performed, inspired by Braun and Clarke’s six-step approach [44, 45]. In the first step, each author read the transcribed interviews thoroughly *to familiarise themselves with the data*. In the second step, the first author (IÅR) generated the *initial codes*. In the third step– *searching for themes*– the first author searched for preliminary themes and merged codes into potential themes. In the fourth step, all the authors discussed and *reviewed these potential themes* in light of the coded data before *agreeing on the final themes* described in the fifth step. The iterative reflection processes within the research group enhanced the validation of the analytical process. For excerpts of the analysis process, see Table 1. In the sixth step, the *results were reported* by presenting the identified themes and using paraphrased text and quotations to illustrate these themes [44].

**Table 1:** Excerpts from the analysis process

Meaning units	Codes	Subthemes	Main themes
[In the academic setting] We saw what the others [fellow students] had written rather than what we had written, in a way. So, of course, people [fellow students] had interpreted it a little differently. Some had written a lot, and others had only written a sentence. There was a big difference in how we interpreted it.	Valuing learning from each other	Appreciation of peer learning during group assignment	Basic learning of EHR documentation
[In the academic setting] I think it was good that we had that assignment and that we got to work with [the academic EHR] and got to do it in groups. We could come up with input and discuss	Importance of discussions with fellow students		
[In clinical placement] I didn’t hear anything [from my contact nurse when I had documented in EHR] Neither did I. But if I had worded it wrong, I think I would have heard something.	Concern that their documentation was not read	Feeling of being ‘on your own’	Experiences and challenges during clinical placement
[In clinical placement] My contact nurse read through what I had written several times and since she didn’t say anything, I assumed that I had formulated the documentation well. I learned and got to know her, so she would have told me if I had written something wrong. But since I didn’t hear anything, I reckon it went well.	Receiving no comments on their documentation		

## Rigour

To enhance trustworthiness, the criteria of credibility, transferability, dependability and confirmability [46, 47] were used. To ensure credibility repeated reflections throughout the analysis process were conducted during meetings among the researchers. All researchers were well-versed in teaching EHR nursing documentation and the first (IÅR) and last author (HS) also had broad experience in qualitative research. Detailed descriptions were sought to enhance transferability. Though the data collection were completed in December 2018, we believe that transferability is of interest to nursing education as knowledge on the topic is still scarce [8, 48] Dependability was pursued by recruiting participants with a high level of information on the subject and by meticulously describing all phases of the research process. Confirmability was based on the perspectives and experiences of the participants, made visible by citations from their focus group discussions when presenting the results. The Consolidated Criteria for Reporting Qualitative Research reporting guidelines checklist was applied to enhance rigour [49].

## Results

The results revealed one overarching theme, two main themes, and six subthemes (Table 2).

**Table 2:** Organisation of the results

Overarching theme	The journey of electronic health record (EHR) learning	
Main themes	Basic learning of EHR documentation	Experiences and challenges during clinical placement
Subthemes	Need for sufficient teacher guidance in an academic setting	Appreciation of mentoring whilst in clinical placement
	Appreciation of peer learning during group assignment	Feeling of being ‘on your own’
		Worrying about patient safety
		Reflection on the two-sided learning process

The overarching theme encapsulated the way students expressed how learning EHR documentation could be experienced as a journey, with continuous reflections on learning during the academic course and clinical placement. The journey displayed practical, intellectual, and emotional characteristics, and some students expressed concerns about whether clinical placements provided appropriate learning.

### Basic learning of EHR documentation

The main theme, basic learning of EHR documentation, encapsulated the perspectives of the students on how learning EHR documentation took place during the academic course.

### Need for sufficient teacher guidance in an academic setting

The students underlined the importance of teacher guidance in the academic course during the three-hour introduction and while working in learning groups. However, they expressed several challenges. The students were satisfied with how the simulated EHR was introduced into the software and how to use the EHR related to a patient case. Despite this, they described the need for more in-depth guidance on explicitly formulating nursing diagnoses, outcomes, and interventions, as the EHR program was based on free-text formulation: ‘It was a good introduction, but I felt perhaps the teaching focused more on how to [technically] create a nursing intervention plan than how we were supposed to formulate the text [in writing]’ (FG1). Several students stated that the teacher/student ratio was approximately 1:25 in the data lab, leading to limited opportunities for individual teacher guidance during their introduction. Students commented that more teachers should be available to provide support at the level and pace needed: ‘…It was quite difficult and challenging to get help [in the data lab] since there was only one person teaching and there were many [students]. There were several [students] who encountered problems’ (FG1). Students also described that the allocated time for guidance was hampered by some students being unprepared for class, which slowed their progress, as many needed additional assistance to proceed.

While working on group assignments, students reiterated the importance of teacher guidance in the learning group. However, they described frustration when, in some instances, the guidance of the teachers was not aligned with what had been presented during the introduction session. One student stated: ‘There was a lot of different feedback from different lecturers’ (FG2). These discrepancies made them insecure about whether or not they were on the right track.

### Appreciation of peer learning during group assignment

Students emphasised the importance of working with their peers during their assignments. Discussions with fellow students enabled them to further understand how to manage the EHR and were important for completing the assignment; as stated: ‘It went well with the EHR documentation’ (FG3). They also described how they conferred across the learning groups, which provided various perspectives on developing a nursing care plan for the EHR. This was described as follows: ‘I think it was good that we […] got to do [the assignment] in groups. So that we could give input and discuss [with other peers].’ (FG3). As such, the importance of peer learning was emphasised.

Some students with previous experience with the EHR software from working as community assistant nurses described that the assignment provided them with an expanded understanding of the EHR compared with that derived from their previous experience, as stated: ‘I have been able to document before and have written interventions […] But the fact that we created a patient account and a [total] nursing care plan [in the academic EHR] has been extremely useful’ (FG3).

### Experiences and challenges during clinical placement

The second main theme, experiences and challenges during clinical placement, encapsulated the perspectives of the students on learning about EHR documentation during clinical placement. Four subthemes were identified.

### Appreciation of mentoring whilst in clinical placement

Students emphasised the importance of guidance provided by the contact nurses in learning how to apply EHR documentation during clinical placement. They described various approaches applied: some of the contact nurses were well prepared; they explained in detail how to effectively use the EHR and they provided concrete feedback on the documentation that the students had conducted. The feedback included how the students could improve, as described by one student:

Then she […] watched while I documented. Then she told me whether I wrote too much, or if there was something more I should write. If I was unsure, I asked her where she would have put it [the data, placement in columns in the EHR]. (FG3)

Another valued approach was how a contact nurse developed a template which was shown and explained to the students at the start of the practice period. The students were able to follow this template when documenting. The student described this nurse as ‘very clever and well experienced’ (FG3). Some students also described how they were encouraged to learn by how different nurses documented. From these various experiences on how to document, students understood the importance of documentation in preparing for possible unforeseen events. The students acquired perspectives on the necessity of well-documented nursing care plans, as stated: ‘I wanted to be ready if unexpected situations occurred, so I felt a need to have a certain overview and control over what might happen’ (FG1). Observing how various nurses documented was described as helpful to students.

### Feeling of being ‘on your own’

Conversely, several students reported that feedback from the contact nurses was scant:

I only wrote alone. But there is a separate code for entering the electronic journal as a student, so you can see from the type of journal that it was written by a student. But I don’t think anyone at all ever looked at what I had written [before] it was saved. (FG2)

Another student expressed a similar experience, hoping it would have been detected if she had written incorrectly: ‘… If I had worded it wrong, I think I would have heard something’ (FG1). Another student described how the guidance appeared somewhat random.

After I asked the first time [about starting to document], I only got “Yes, then you can go and write”, and then, I didn’t know where I was supposed to do things. But then I told them that I had made a mistake, and they probably realised that I had to have some guidance [in the EHR], so I got it [guidance] after that, at least a little bit. (FG2)

Some students also described how they felt they were expected to manage the EHR from the beginning of their clinical placement, as exemplified by this quote:

For me, it was very much like […the nurses told me] “Yeah, just go and write”, so […] it was almost like that from day one, so I felt […] that I was a bit uncertain at the start and that I did not receive much help, that they just expected me to do it. (FG2)

Variations in how much the students were able to document were also described. Some made daily EHR entries, adjusting their care plans and registering lab values. Others were told that documentation was recorded only when there was a change in the patient situation. This meant few opportunities to learn EHR documentation for one student: ‘I documented maybe three times [in the EHR] during six weeks’ (FG3).

### Worrying about patient safety

Some students described that the healthcare workers often did not read the EHR:

I have a general impression that in the unit where I was, there was very little reading of the health records. That is, people were very good at writing, but no one read what had been written; it seemed like, unless there was something special […] there was no one who, on a normal day, saw how the patient had been doing regarding care and food; I don’t think anyone looked at that. I think it was just stored and left there. (FG2)

At several clinical placements, the students noticed that the EHRs were not updated, making them deeply concerned about patient safety, as stated: ‘And then there were written assessments, but they were from about a year ago, so they had not been updated since then’ (FG3). Some students compared their other work experiences, where documentation routines were strict, and wondered why such practices were not implemented in their clinical placements. Another student worried whether a patient was malnourished but was unable to find any documentation on height or weight to calculate the body mass index:

Yes, it was quite extreme because we had some [patients] who were malnourished; I thought, “How do you make sure that patients gain weight? How do you check that?” So, […] you might be able to see it [visually observing the patient], but you can’t see it right away. In other words, do document, but then nothing is documented [in the EHR]. It’s absolutely horrible. (FG3)

The students were also concerned about patient safety with regard to insufficient documentation routines and inconsistencies in documentation, as documentation was carried out both on paper and in the EHR: ‘As it was not updated [in the EHR], everything had to be on paper, as if we are in 1914’ (FG3). Another quote addressing this issue was: ‘I experienced a bit of a mixture. They sort of documented “ate according to plan,” but the plan was a piece of paper hanging on a kitchen cupboard. So, like, “compared to which plan”?’ (FG3).

Some students found the verbal report provided between shifts to have more information than that written in the EHR. This occurrence made the students wonder and relate this incident to what they had learned in the academic setting about the requirements of documenting nursing care: ‘I think if it’s important enough to give [a verbal] report, then it’s also important to write it [in the EHR], this is at least what we have learned’ (FG1). Some students also reflected on whether patients with physical disabilities received disproportionately more comprehensive documentation.

However, the students noted that when a new patient was introduced to the unit, the EHR was completed as they had learned in the academic setting: ‘… [when we] got a new patient in, we had to create new data collection and care plan […] and then it was used, the things that we learned in school’ (FG3).

### Reflection on the two-sided learning process

Students described the academic EHR course as relevant to executing the EHR documentation in their clinical placement. As described by this student:

It helps to have been through it ahead, even if you don’t remember the details of everything that can be done [in the EHR program]. It helps to have seen the different screens and makes it easier to familiarise yourself with it again when you really need to use it [in practice]. (FG2)

However, as the students had various experiences regarding how well healthcare workers executed the EHR during the clinical placement, they also reflected on the importance of being taught this at university in advance. As described by one student, the academic course provided them with knowledge and training on how EHRs should be executed.

… as you enter the clinical placement and see what they [healthcare workers] have done [documented], you realise that what they have done is wrong. So, you kind of don’t learn from them, as you have already learned how it actually should be done […]. It is a good thing we have this [the EHR course] in advance. (FG3)

The students expressed concern as to whether they could have been misdirected if their only reference on how to apply EHRs had been the practice provided during their respective clinical placements, as stated: ‘In other words, if you were to start learning it now [in clinical placement], we could have been taught wrongly’ (FG3). Learning acquired during the academic course functioned as a compass, guiding students through rugged terrain.

To improve the relevance of the compulsory course for the upcoming clinical placement, students suggested including several parts of the EHR program, exemplified by how to write reports and record deviations, as these were areas they also documented in practice. Those students who had completed the course several weeks before the clinical placement suggested, for optimal preparedness, a refresher course closer to the start of the placement period.

## Discussion

A major finding of this study was how students underlined the value of feedback during their journey to learn about EHR documentation. Students addressed the importance of feedback from teachers and peers in the academic setting and from contact nurses during clinical placement; all important insights providing meaningful responses to enhance EHR documentation learning. Feedback enhances student learning [24, 25] and timely, constructive, and considerate feedback is important [25]. In clinical settings, mentor responses should be prompt, well-structured, and tailored to the individual student [24]. In the context of peer learning and feedback, this approach aligns with the student-centred pedagogical approach promoted in higher education [26]. Peer feedback can enhance language proficiency and clarity of expression while also serving as a tool for brainstorm and refining reasoning processes, thereby providing alternative perspectives, ideas, and insights [50]. These are essential factors in the development of the clinical competence of nursing students and are connected to their ability to apply knowledge from an academic to a clinical setting [24]. Divergent thinking can contribute to developing a high-quality nursing care plan within the EHR. Feelings of not being supported in the classroom, lab or clinical environment influenced the confidence of students in forming theory-to-practice connections [51]. The importance of emphasising qualified feedback in all settings and by various stakeholders when implementing EHR documentation in nursing education was highlighted in our study.

However, our study revealed variations in how contact nurses provided EHR support during clinical placements. Some students worked with knowledgeable and skilled nurses who guided them with expertise. Together, the contact nurse and students reflected on the EHR documentation which the students had furnished, and the contact nurse provided concrete feedback on how they could improve. Students described how this enhanced their learning. This aligns with Vygotsky’s theory on ZPD when a more skilled person can enable an unskilled person to stretch towards their learning limits [23]. The nurses, being more skilled persons, promoted student learning by purposefully outlining and expanding their learning potential. On the contrary, students in our study also felt they were left to document independently and received little feedback on whether their documentation was sufficient. The limited application of this crucial pedagogical approach may warrant reflection on the pedagogical competencies needed when guiding students. Official Norwegian regulations require academic nursing education to offer training in clinical supervision. However, there are no stipulations for contact nurses to have the pedagogical qualifications to become contact nurses [5], despite the importance of such competencies for ensuring quality in clinical practice supervision [25]. Supervisors are important facilitators who help students apply what is learned at university to bridge the gap between theory and practice [20]. Cognitive dissonance may occur when students experience a conflict between what they have learned at university and what is experienced in clinical placement. This dissonance may lead to stress, anxiety, and feelings of incompetence [20], as the students struggle to determine whether their performance and delivery aligns with what is expected [8].

Being in the first year of their program, the nursing students in our study underlined the importance of being introduced to the EHR in the academic setting through a teacher-led introduction at the data lab and during subsequent work in peer groups. Several studies have highlighted the introduction of simulated EHRs in academic settings at an early stage of education [15, 19, 22]. Using simulated EHR ahead of clinical placement intensified student confidence when using EHR during clinical placement [16]. A recent review emphasised SBL as enhancing theory preparedness and transition and improving self-reported confidence and competence during clinical placement [21]. Early exposure to the EHR has been emphasised to promote self-efficacy and competence in nursing informatics and clinical reasoning in nursing education [22].

One disturbing result in our study was how students described their uneasiness and major concerns regarding insufficient EHR documentation. They were concerned about patient safety being at risk, resulting in disquietude and anxiety. Owing to some units’ lack of consistency between what was recorded on paper and in the EHR, and that nursing care plans in the EHR were not being updated, students worried that failures could arise. These results address the issue of the learning quality in the clinical placements. Given the central role of clinical training in nursing education, assessing the quality of such internships is crucial [52]. Evaluating the clinical environment is important for enhancing learning experiences, including those related to EHR documentation. The growing development of psychometric tested evaluation forms for clinical learning environment, may reflect the increasing emphasis on ensuring quality in clinical placement [53].

The students in our study underlined how the academic course provided them with a foundation that was crucial when they experienced insufficient guidance in the clinical setting. Learning EHR documentation in an academic setting provided students with qualified standards that were important to have acquired in the event of malpractice. Academic course content served as the benchmark. Theories on the transfer of learning draw attention to the alignment between academic settings and practice. When there is incongruence between classroom and practice, students struggle to link theory to practice [51]. Thus, the results of this study provide a more nuanced perspective. The students identified discrepancies and, to a large extent, had misgivings as to whether they would have adopted this malpractice without having been furnished with adequate provisions from the academic EHR course. The benchmark learned in the academic EHR course provided confidence in their knowledge of how EHR documentation should be provided and executed. Evidence-based practice is found to be important in bridging the theory-practice gap [20]. Academic EHR learning, using simulated evidence-based practice, may safeguard students against faulty learning in situations where malpractice is encountered.

While studies sharing expertise and strategies among educators is increasing, it still remains relatively scarce [9, 17]. Incorporating students’ voices in such research is encouraged [29, 30] However, further research is needed to explore the impact of academic EHR training on its implementation during clinical placements.

### Limitations

It is important to note that although the students had clinical placements in six different nursing homes, the conclusions in this study are based on the experiences and observations of students from a single university context. Therefore, to enhance transferability to other contexts or environments, further research is needed. Since the data was collected in 2018, significant changes may have occurred, highlighting the need for further research to provide insight into the current situation. Transcripts and the analysed data were not returned for validation by the participants [49] owing to practical factors.

## Conclusion

The study highlighted the significance of teaching EHR documentation in an academic setting and revealed differences in students learning of EHR documentation during clinical placements. The study emphasized the crucial role of feedback in the learning process. It also indicated that EHR documentation education should be enhanced, especially in clinical settings, as the students reported receiving insufficient feedback during clinical placements, raising concerns about patient safety. Thus, the study demonstrated the need to improve the quality and quantity of feedback from both teachers and contact nurses, to enable students to effectively learn and apply documentation. Additionally, the research emphasized the importance of academic instruction as a robust foundation that safeguarded students from potentially adopting incorrect documentation practices in clinical settings.

## Electronic supplementary material

Below is the link to the electronic supplementary material.

Supplementary Material 1

## Data Availability

Data supporting the findings of this study are available upon request from the corresponding author. The data are not publicly available due to privacy and ethical restrictions.

## Abbreviations

ECTS: European Credit Transfer and Accumulation System
EHR: Electronic health record
GDPR: General Data Protection Regulation
NSD: The Norwegian Social Centre for Research Data (from 2022 called Sikt, The Norwegian Agency for Shared Services in Education and Research)
SBL: Simulation-based learning
WHO: World Health Organization
