# Supplementary material for: Nursing students’ perspectives on learning electronic health record documentation in an academic setting and during clinical placement: a qualitative study
Source: BMC Nurs. 2025 Jul 1;24:764. doi: 10.1186/s12912-025-03320-5 (PMC12211296; doi:10.1186/s12912-025-03320-5)
Supplement: Supplementary file 1 — Supplementary Material 1 [file 12912_2025_3320_MOESM1_ESM.docx]

**Supplementary File 1. Interview guide**

This study aimed to explore the perspectives of first-year bachelor’s degree nursing students on learning electronic health record (EHR) documentation in an academic setting and during their first clinical placement.

**On campus - at university:**

1) Did you participate in the EHR course?

2) In what way do you think the EHR course is relevant to your internship?

**Clinical placement:**

3) How was the EHR system used in your clinical placement?

4) How did you use the EHR?

5) If you did not use the EHR, what was the reason?

**Contact nurse – supervisor:**

6) Did you receive guidance from the contact nurse?

7) How did the contact nurse contribute with guidance?

**Evaluation:**

8) How would you evaluate your own EHR application?

9) How do you evaluate the use of EHR during your clinical placement?

10) Regarding teaching EHR at university and supervision during internship: In hindsight, what could have been different, and what could be improved?
